# Combined Spinal Epidural Anaesthesia with BiPAP-Three Case Reports

**Published:** 2009-08

**Authors:** Ashok Jadon, Neelam Sinha, Prashant S Agarwal

**Affiliations:** 1 Senior Consultant and Head, Department of Anaesthesia, Tata Motors Hospital, Jamshedpur-831001, Jharkhand, (India); 2,3 P.G. Student, Department of Anaesthesia, Tata Motors Hospital, Jamshedpur-831001, Jharkhand, (India)

**Keywords:** BiPAP, COPD, CSEA, Hypoventilation, Laparoscopic cholecystectomy, Propofol sedation

## Abstract

**Summary:**

We report three cases where BiPAP (bi-level positive airway pressure) was used with CSEA (combined spinal epidural anaesthesia) to over come the hypoventilation due to preoperative poor respiratory reserves and additive effect of sedation. Combination of BiPAP with spinal, epidural and CSEA have been used successfully in patients of severe COPD (chronic obstructive pulmonary disease) for various surgical procedures. This combination provides safe alternative to conventional general anaesthesia, as it avoids need for postoperative ventilatory support and its deleterious effects.

## Introduction

Severe chronic obstructive pulmonary disease (COPD) cases for surgery carry high risk of perioperative morbidity and mortality due to poor respiratory reserve, and associated systemic diseases like hypertension, corpulmonale, CCF (congestive cardiac failure) etc. General anaesthesia if possible, is better avoided due to risk of impending respiratory failure and need for postoperative ventilatory support.^1^,^2^ Spinal and epidural anaesthesia provides safe and effective anaesthesia in such high riskpatients.^3^ But problem of intraoperative sedation remain unsolved as sedation may cause hypoxia especially in anxious patients who want to be unconscious and, for laparoscopic procedures where sedation is required to avoid the discomfort of C02 insufflation. Upper abdominal operation requires adequate analgesia up to T4 which always compromise on respiratory muscle functions and when sedation is given in already respiratory compromised patients hypoxia is inevitable. However, this hypoxia can be prevented by using intraoperative BiPAP, as it supports the patient's own respiration without interfering airways and preventing hypoxia by maintaining functional residual capacity(FRC). This concept recently has been used in compromised respiratory system patients of severe COPD for various surgical indications.^4^,^5^ We report the use of a combination of combined spinal epidural anaesthesia (CSEA) and bilevel positive airway pressure(BiPAP) in three patients of severe COPD for inguinal hernia repair, laparoscopic chole-cystectomy and radical hysterectomy.

## Case-1

An 82-yr-male patient presented with obstructed right inguunal hernia. He was a known case of advanced COPD, corpulmonale and pulmonary artery hypertension. He had very poor respiratory reserve, he was confined to bed with oxygen support at most of the time of the day, and he was normally unable to lie flat. He had many episodes of CCF and hospitalization in intensive care. Echocardiography showed mild aortic regurgitation with decreased left ventricular function. ECG showed ST depression in inferior leads. Blood investigations and electrolytes were normal.

## Case-2

A 65-yr-female patient presented with gall stones and scheduled for laparoscopic cholecystectomy. She was confined to bed and was under treatment for paraplegia for 2 months, MRI showed compression at D7. She was a case of COPD, old pulmonary tuberculosis, NIDDM on oral hypoglycemic, ischemic heart disease with recurrent chest pain, ECG showed left bundle branch block(LBBB), old anteroseptal infarction with left axis deviation. Echocardiography showed thin and hypokinetic intraventricular septum, mild LV systolic dysfunction and 44% left ventricular ejection fraction. She had history of untoward cardio-respiratory event under general anaesthesia and intensive care admission (details not available, procedure was abandoned) during Endoscopic Retrograde Cholangiopancreatography (ERCP) for common bile duct (CBD) stent, two weeks before in other hospital.

## Patient-3

**Fig 1:**
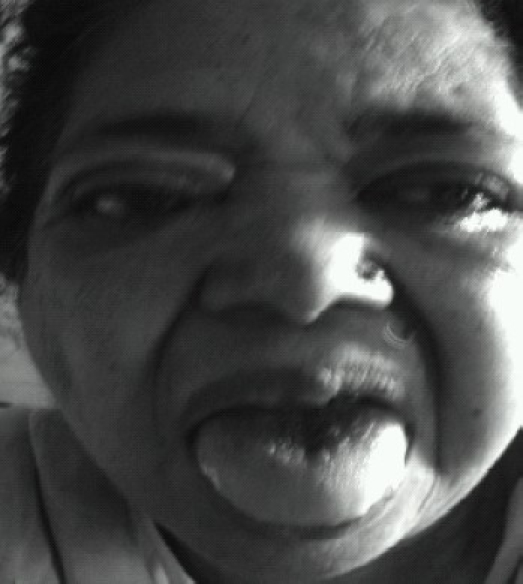
Photograph of patient #3 with difficult airway (Mallampati class-4) (Consent for Photograph and publication taken)

A 70 yr, 86 kg female patient (Fig 1) scheduled for radical hysterectomy. She was a known case of hypertension, diabetes mellitus and COPD and episodes of sleep apnea. She was obese, had difficult airways (MPS 4) and had history of difficulty in maintaining airways under general anaesthesia (midazolam + propofol + sevoflurane) in last surgery for cervical biopsy 7 days before in our hospital which was managed with bag and mask oxygenation by two anaesthetists.

## Anaesthesia technique

Informed consent for high risk was taken from patients, possible optimization of general condition (antibiotics, insulin, bronchodilators etc.) was done, and medicines were continued as indicated in preoperative period. In operation theatre standard monitoring was commenced, i.v access established, and an i.v. infusion of normal saline solution started. Oxygen was administered through nasal prongs. Combined spinal epidural technique by needle through needle (CSE Cure, Portex Combined Spinal/Epidural minipack 27G/18G) was used for anaesthesia. Epidural catheter (18G) was inserted through Tuohy needle 3-4cm in epidural space and after negative aspiration test for blood and CSF 2ml saline was used to flush the catheter to know the patency. Level of block was decided by nature of operation and epidural top-up were given as required (Table-1). Sedation was given when patient requested for sleep or showed undue anxious and uncooperative behavior. Initially with 0.5mg increments of midazolam and 10-20mg bolus of propofol and then infusion of propofol was started @ 0.5mg/kg/hr. BiPAP (BiPAP® Auto-M Series RESPIRONICS®) was started when SpO2 did not improve with oxygen by nasal prongs or Poly mask In first two patients IPAP-14 and EPAP-5 adequately maintained oxygenation, in hysterectomy patient IPAP-20 was required when Sp02 did not improve above 87%. ABG was done after one hour of BiPAP commencement (Table-2). BiPAP was gradually with drawn (depending up on patients' acceptance) and oxygen was continued by Poly mask in postoperative period. Postoperative analgesia was provided with 6m1 epidural injection of 0.125% bupivacaine + buprenorphine 100-300 μg on demand basis. All three patients had uneventful recovery and discharged from the hospital.

**Table 1:** Level of CSEA, duration and nature of surgery, amount of spinal and epidural drugs and settings of BiPAP

Operative procedures	Level of CSEA	Dose of 0.5% heavy bupivacaine for spinal	First Dose of epidural lidocaine 2% with adrenaline	Epidural Top-ups lidocaine 2% with adrenaline	Duration of surgery (minutes)	IPAP	EPAP
Inguinal hernia repair (bilateral)	L3/L4	1.0ml	5.0ml	10ml	156	14	05
Laparoscopic cholecystectomy	T9/T10	2.0ml	10.0ml/10ml+	10ml	160	14	05
Radical hysterectomy	L2/L3	3.0ml	3.0ml	17ml+/15ml	190	20	06

**Table 2:** ABG values after one hour of BiPAP application with 3 L/min Oxygen

Patients	PO2mmHg	PCO2mmHg	PH	SaO2%	HCO_3_ mEq/l
Inguinal hernia repair (bilateral)	86.1	38.1	7.43	95.9	34
Laparoscopiccholecystectomy	107	45	7.414	98.9	27
Radical hysterectomy	88	36.1	7.35	95.9	22

## Discussion

Spinal and epidural anaesthesia are beneficial for both obese and advanced COPD patients. Compared with general anaesthesia, the maintenance of spontaneous breathing means there is less cephalad displacement of the diaphragm and less risk of atelectasis, closing capacity and FRC are less affected and pulmonary gas exchange is better maintained.^3^ However, sedation given in conjunction with a regional block decreases sensitivity to C02 and hypoxia, and thus these patients are unable to deal effectively with hypercarbia and hypoxia moreover, combined effect of pneumoperitoneum (as in laparoscopic cholecystectomy) and sedation can lead to hypoventilation and arterial oxygen desaturation.^6^ Superior postoperative analgesia without risking respiratory depression, and avoidance of the strong stimulation of intubation or the risk of broncho constriction on extubation, all of these benefits have been reported in the use of combined spinal and epidural anaesthesia for abdominal aortic aneurysm repair in patients with severe COPD.^5^

**Fig 2:**
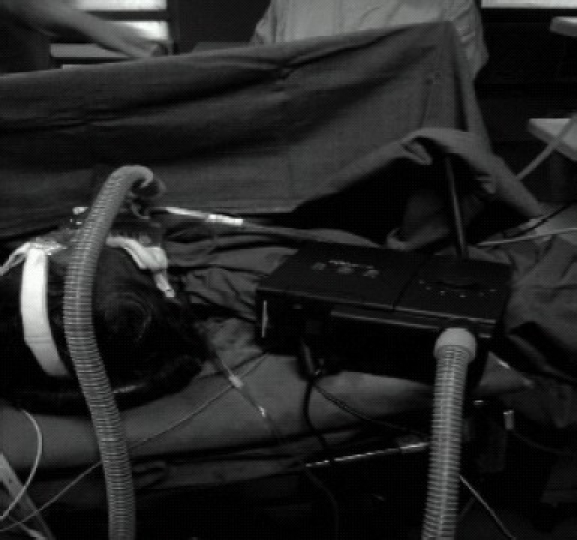
Patient#2 (laparoscopic cholecystectomy) showing BiPAP machine, oxygen source and protective eye pads

We used combination of BiPAP(Bi-level positive airway pressure) and combined spinal epidural anaesthesia (CSEA) in our three high risk patients scheduled for inguinal hernia rep air, laparoscopic cholecystectomy and hysterectomy having multiple systemic diseases including poor respiratory reserves due to severe COPD. CSEA is a better option in high risk patients because, it provides safe and effective neuraxial block than either spinal or epidural alone.^7^ BiPAP helped to maintain oxygenation (Table-2) when patients were sedated with propofol and were unable to maintain oxygenation^8^ with conventional methods e.g. nasalprong and Poly mask. General anaesthesia could have been an alternative with intubation and IPPV but there was likelihood that these patients would need postoperative ventilation and, general anaesthesia it self has detrimental effects on postoperative respiratory functions.^3^,^6^ Noninvasive ventilation and propofol sedation with spinal, epidural and CSEA has been used and accepted clinically practicable method in various surgical procedures and it helps to correct alveolar hypoventilation during spinal anaesthesia,^4^,^5^,^8^,^9^ There are complications associated with the use of non-invasive positive pressure ventilation (NIPPV) and these include local trauma, gastric distension, eye irritation, sinus congestion, air leaks, and haemodynamic effects.^4^ These problems were managed with protective eye pads (Fig 2), nasogastric tube (some time this interfere with airtight seal), extended neck position and selecting lower BiPAP values (IPAP-14 and 20, EPAP 5-6) and intra-venous fluids.

The use of BiPAP from beginning of procedure and in a planed manner is ideal to avoid poor patient compliance. This is achieved by a controlled, gradual introduction, checking the patient's acceptance before performing the spinal, and then the use of target controlled sedation during surgery.^4^

We report the use of a combination of combined spinal epidural anaesthesia and BiPAP (bi-level positive airway pressure) in three patients of severe COPD who developed hypoventilation when sedation was given This technique helps in managing high risk COPD patients with advanced lung disease who are at risk of hypoventilation due to sedation under regional anaesthesia.
